# Neighbourhood characteristics and social isolation of people with psychosis: a multi-site cross-sectional study

**DOI:** 10.1007/s00127-021-02190-x

**Published:** 2021-11-17

**Authors:** Domenico Giacco, James B. Kirkbride, Anna O. Ermakova, Martin Webber, Penny Xanthopoulou, Stefan Priebe

**Affiliations:** 1 Division of Health Sciences, Warwick Medical School, University of Warwick, Gibbet Hill Campus, Coventry, CV4 7AL England; 2 Unit for Social and Community Psychiatry, (WHO Collaborating Centre for Mental Health Service Development), Barts and the London School of Medicine, Queen Mary University of London, Newham Centre for Mental Health, London, E13 8SP England; 3 Division of Psychiatry, University College London, London, W1T 7BN England; 4 Department of Life Sciences, Faculty of Natural Sciences, Imperial College London, Silwood Park, Buckhurst Road, Ascot, Berks SL5 7PY England; 5 Department of Social Policy and Social Work, University of York, Heslington, YO10 5DD York UK; 6 College of Medicine and Health, University of Exeter, St Luke’s Campus, Exeter, EX2 4TH UK; 7 Present Address: Coventry and Warwickshire Partnership NHS Trust, Coventry, England

**Keywords:** Social isolation, Population density, Social deprivation, Social fragmentation, Schizophrenia

## Abstract

**Purpose:**

People with psychosis are vulnerable to social isolation, which is associated with worse clinical outcomes. In general populations, people living in areas with higher population density have more social contacts, while those living in more socially deprived and fragmented areas are less satisfied with their relationships. We assessed whether and how neighbourhood factors are associated with social contacts and satisfaction with friendships for people with psychosis.

**Methods:**

We carried out a cross-sectional study including people with psychosis aged 18–65 years in urban and rural sites in England. Population density and social deprivation and fragmentation indexes were described within Lower Level Super Output Areas (LSOA). Their associations with participants’ social contacts and satisfaction with friendships were tested with negative binomial and ordinal regression models, respectively.

**Results:**

We surveyed 511 participants with psychotic disorders. They had a median of two social contacts in the previous week (interquartile range [IQR] = 1–4), and rated satisfaction with friendships as 5 out of 7 (Manchester Short Assessment of Quality of Life; IQR = 4–6). Higher population density was associated with fewer social contacts (Z-standardised relative risk [RR] = 0.88; 95% CI = 0.79–0.99, *p* = 0.03), but not with satisfaction with friendships (RR = 1.08; 95% CI = 0.93–1.26, *p* = 0.31). No associations were found for social contacts or satisfaction with friendships with social deprivation or fragmentation indexes.

**Conclusions:**

Clinicians in urban areas should be aware that their patients with psychosis are more socially isolated when more people live around them, and this could impact their clinical outcomes. These findings may inform housing programmes.

**Supplementary Information:**

The online version contains supplementary material available at 10.1007/s00127-021-02190-x.

## Introduction

Social isolation is a predictor of early mortality and of poor physical and psychological health outcomes in the general population [1–3]. It is, therefore, a serious clinical concern for people with psychotic disorders who have, on average, fewer social contacts [4, 5] and are less satisfied with their social relationships than the general population and other people with mental or physical health conditions [6–8]. Higher levels of social isolation are linked with more severe symptoms [6, 9] and higher use of inpatient services [10]. Social support has been found to facilitate recovery from psychosis from patient perspective [7].

Vulnerability to social isolation can be partially explained by individual-level variables, such as more severe symptomatology [6–8, 11], unemployment or single marital status [4], which predict some of the differences in subjective (e.g. satisfaction with social relations, loneliness) and objective (e.g. social network size, number of social contacts in a specific timeframe) indicators of social isolation. However, a large amount of variation in measures of social isolation amongst people with psychosis remains unexplained.

One possibility is that the wider social environment of a person experiencing psychosis may affect their degree of social isolation as the environment is strongly linked with other aspects of psychosis. For example, areas with higher levels of social deprivation, fragmentation (i.e., the absence of connections between individuals and society), and population density have higher incidence rates of psychotic disorders [12–15]. Furthermore, the use of services by people with severe mental illness appears to be greater in areas with higher social deprivation [16, 17], while an increase in population density is linked to lower hospitalisation rates [16].

To identify whether there was any evidence of associations between neighbourhood-level characteristics and subjective and objective indicators of social isolation, we carried out a systematic appraisal of the literature (see box 1 for methodology used). We identified six cross-sectional studies of general populations (non-clinical samples). Three studies [18–20] reported that the number of social contacts increased in areas with higher social propinquity (i.e., physical or psychological proximity), and one study found that people living in more densely populated areas had more social contacts, even if not necessarily with neighbours [21]. Two studies showed that higher social deprivation and fragmentation were associated with subjective aspects of social isolation, such as reduced social trust [22] and greater loneliness [23].

Our systematic search did not identify any studies assessing the associations of neighbourhood-level characteristics and social contacts of people with psychotic disorders, despite their vulnerability to social isolation [4, 6].

Box 1: background literature search—methods We searched EMBASE, MEDLINE, and Web of Science for studies published in any language. The review was carried out as a background of this work and later updated to March 31, 2020. Our search terms were "neighbourhood" OR "social deprivation" OR "social fragmentation" OR "population density" AND "social contacts" OR "social isolation”. We also screened references of reviews in related areas.

## Aims of the study

In this study, we assessed the relationship between neighbourhood-level factors and objective (social contacts involving at least a brief conversation) and subjective (satisfaction with friendships) indicators of social isolation.

## Materials and methods

### Study design and participants

In the period between the beginning of June 2017 and the end of May 2018, we conducted a cross-sectional survey in community mental health teams across six participating NHS Trusts covering a range of geographical areas, in both urban and rural contexts: Cornwall Partnership NHS Foundation Trust; Devon Partnership NHS Trust; East London NHS Foundation Trust (covering East London, Luton and Bedfordshire); Oxford Health NHS Foundation Trust (covering large areas of Oxfordshire and Buckinghamshire), and Somerset Partnership NHS Foundation Trust; Tees, Esk and Wear Valleys NHS Foundation Trust (covering county Durham, Darlington, Teeside and North Yorkshire). Participants were identified from secondary mental health care service caseloads from clinicians or clinical study officers.

Participants were included if they conformed to the following conditions: were aged 18–65 years; had a clinical diagnosis of a psychotic disorder according to the International Classification of Disease-10 (ICD-10) codes F20-29, as identified in clinical records; were receiving care from outpatient secondary mental health services or primary care services; had capacity to provide informed consent; and were able to communicate in English. Participants were excluded if they had a current and primary diagnosis of substance use disorder (ICD-10, F10-19), had been hospitalised in the previous week (although these potential participants could be re-approached at a later time), or their postcodes could not be obtained because they were homeless or living in temporary accommodation at the time of the survey. All participants provided written informed consent.

### Procedures and measures

Eligible participants were identified by members of their wider clinical team and asked for their consent to speak to a researcher. Participants then completed the study questionnaires at the presence of the researcher. Participants could either complete the questionnaire themselves or ask the researcher to read out the questions for them and complete the questionnaires, based on their verbal instructions. Researchers also obtained consent to access participant clinical records to retrieve clinical and socio-demographic characteristics. Data was entered into a database held on a secure server.

The questionnaire asked participants to self-report on two measures. First, using the Social Contacts Assessment (SCA) [24], participants reported the number of social contacts in the previous week. According to the SCA, a “social contact” was someone the participants could name and with whom they would have had at least a brief face-to-face conversation (more than just greeting) in the last week. Participants were asked not to include people they were living with or mental healthcare professionals. For employed participants, people they worked with could only be included if contacts took place outside their workplace and were not related to their work. This will be referred to as an “objective measure of social contacts” as, whilst influenced by the recall and personal appraisal of social contacts of a participant, refers to contacts which have actually happened in the previous week.

The Social Contacts Assessment (SCA) is a questionnaire aimed to count social contacts in the previous week. This was used previously in an observational study [24] and a randomised controlled trial in England [25]. Its use so far showed ease of completion by participants and sensitivity to change.

The SCA is enclosed as Appendix I in the online supplementary material.

Second, participants reported satisfaction with the quality and quantity of friendships, measured using the sixth item of the Manchester Short Assessment of Quality of Life (MANSA), i.e. ‘how satisfied are you with the number and quality of your friendships’ [26], which was rated on a score from 1 (very dissatisfied) to 7 (very satisfied). This will be referred to as a “subjective measure of social contacts”, as it assesses a subjective appraisal of social contacts and friendships which may be somewhat independent from the frequency or recency of social contacts.

The MANSA is a widely used questionnaire to assess quality of life of people with severe mental illness throughout the world. It has been validated in both the United Kingdom [26] and elsewhere [27].

We also collected additional participant characteristics such as age, gender (male/female), marital status (single/in a relationship), country of birth (born in the United Kingdom/born in a different country), education level (tertiary or higher/lower), living situation (living alone/not living alone), accommodation (living independently/living in supported accommodation), employment (employed/not employed), receipt of welfare benefits (or not), and length of illness (calculated in number of years from the day of first contact with mental health services). These were collected from participants’ assessments and checked against available data in medical records.

To collect data about the residences of participants, we used Lower Layer Super Output Areas (LSOA)—defined as a small geospatial statistical unit used in the UK Census with a minimum population of 1000 and an average of 1500 designed to improve the reporting of small area statistics in England and Wales [28]. LSOA were obtained from postcodes for participants' current address at the point of assessment. To ensure confidentiality, postcodes were not stored in our database.

Neighbourhood-level characteristics of population density, index of multiple deprivation, and social fragmentation index were derived from UK 2011 Census data [28]. Population density was defined as the number of usual residents per hectare, a metric unit of area defined as 10,000 square metres or approximately 2.47 acres. The population density score was Z-standardised. The Index of Multiple Deprivation (IMD) is the official measure of relative deprivation for small areas (neighbourhoods) in England. It draws on multiple sets of data to estimate an overall rank for deprivation across several domains (income, employment, education, health, crime, barriers to services, housing quality) We used IMD scores linked to each participant’s LSOA of residence from the 2011 Indices of Deprivation [29]. The Social Fragmentation Index (SFI) aims to capture aspects of the local population that may reflect a greater collective risk of social fragmentation/lack of social cohesion. The index is built from four census variables, based on the proportion of the relevant resident population/households who were: (a) unmarried persons; (b) single-person households; (c) privately rented households; (d) living at a different address in the previous year (residential mobility). IMD and SFI were Z-standardised and summed, with higher scores indicating more social fragmentation.

### Statistical analysis

Descriptive statistics (i.e., median and interquartile range [IQR]) were reported for the number of social contacts in the previous week, score of satisfaction with friendships, neighbourhood-level variables, and the socio-demographic and clinical variables described above.

One variable (length of illness) showed a higher percentage of missing values than our a-priori threshold value (5%). Hence, multiple imputation by chained equation was used for all missing values, using all variables included in the analysis as the basis for imputation. All values of regression analyses are presented as pooled estimates following five rounds of multiple imputation procedures.

Two separate regression models were fit, which had two different outcome variables, i.e., the number of social contacts in the previous week and the satisfaction with friendships score. We treated the number of social contacts as a count variable, modelled using negative binomial regression given the evidence of overdispersion in our data (mean = 2.9, variance = 6.9). Our second variable (satisfaction with friendships) was ordinal, hence we used an ordinal regression to model this data.

Modelling for both variables was exploratory and proceeded as follows. Univariable a priori association of outcome variables of regression models with neighbourhood-level variables and participant-level variables were tested. If an association at the level of *p* < 0.05 for neighbourhood-level characteristics (main independent variables) and at the level of *p* < 0.10 participant-level characteristics (covariates) was found in univariable models, these variables were then added to the final multivariable models.

Sensitivity analyses were carried out using only complete cases and are provided in the online appendix as supplementary material. No differences in findings compared to the primary analysis were present.

All multivariable regression models were set at a significance level of *p* < 0.05. We reported relative risk estimates for the association between neighbourhood-level variables and number of social contacts, and estimates from the ordinal regression model for the change in satisfaction scores, along with their 95% confidence intervals (95% CI). Analyses were carried out with the Statistical Package for the Social Sciences (SPSS), version 26.0 [30].

### Role of the funding source

The funders of the study had no role in study design, data collection, data analysis, data interpretation, or writing of the report. The corresponding author had full access to all the data in the study and had final responsibility for the decision to submit for publication.

## Results

**Fig. 1:**
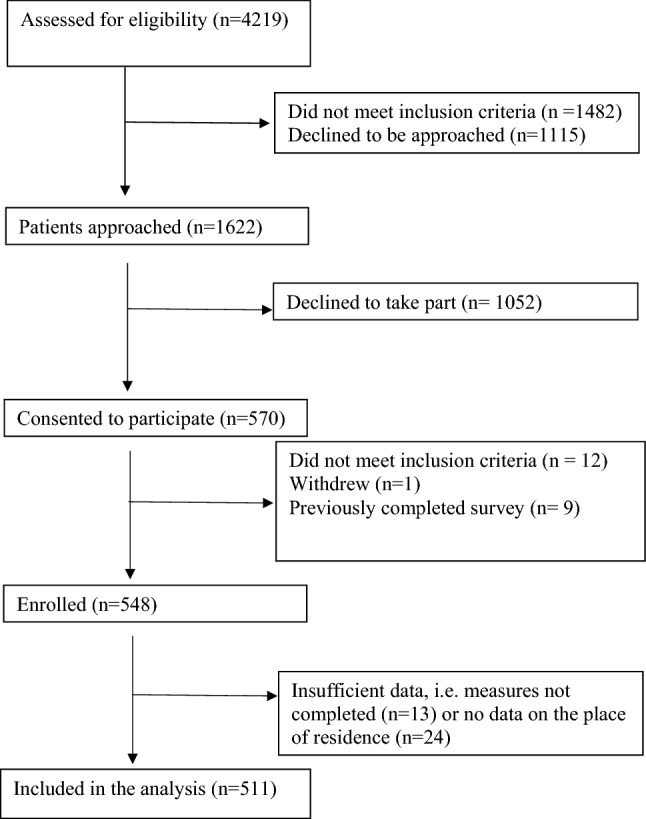
CONSORT diagram

Inclusion criteria were met by 511 participants who were living in 390 LSOA (Fig. 1), the median of participants per LSOA was 1, and there were a maximum of four participants per LSOA. There were no missing cases for the number of online social contacts, the population density index, the social fragmentation index and the index of multiple deprivation.

Data on satisfaction with friendships were missing for nine participants, 1.8% of the whole sample and available for 502 participants (out of 511). For all variables but one (length of illness), missing values were less than 3%. For the length of illness, missing values were 11.8% of the total cases. All missing values were replaced via multiple imputation techniques, as described above. The median age at recruitment was 44 years (IQR 36–52), 178 (34.8%) participants were female, and 394 (77.1%) of the participants were born in the United Kingdom (Table 1). The median number of social contacts in the previous week was 2 (IQR = 1–4) with a median score of satisfaction with quality and quantity of friendships of 5 out of 7, IQR = 4–6). Median population density was 50.3 people per hectare (IQR = 23.3–112.8) and median values for the IMD and SFI were 29.2 (IQR = 16.8–39.8) and 1.8 (IQR = − 0.3 to − 4.2), respectively (Table 1).

**Table 1:** Socio-demographic and clinical variables

Neighbourhood-level characteristics*	
Population density, people per hectare, median (interquartile range, IQR)	50.3 (23.3–112.8)
Index of Multiple Deprivation score, median (IQR)	29.2 (16.8–39.8)
Social Fragmentation Index score, median (IQR)	1.8 (− 0.3 to 4.2)
Participant-level characteristics	
Age, median (IQR)	44 (36–52)
Gender, female, *N* (%)	178 (34.8)
Marital status, single, *N* (%)	383 (75)
Born in the United Kingdom, *N* (%)	394 (77.1)
Education level	
Primary, *N* (%)	37 (7.2)
Secondary, *N* (%)	215 (42.1)
Tertiary/Further education, *N* (%)	246 (48.1)
Living situation, living alone, *n* (%)	236 (46.2)
Living in independent/unsupervised accommodation, *N* (%)	380 (74.4)
Any employment (full-time, part-time, voluntary or sheltered), *N* (%)	101 (19.8)
Receiving state benefits, *N* (%)	489 (89)
Years since first contact with mental health services, median, (IQR)	17 (10–24)
Diagnosis	
Schizophrenia, *N* (%)	250 (68.5)
Schizotypal disorder, *N* (%)	3 (0.6)
Delusional disorder, *N* (%)	12 (2.3)
Brief psychotic disorder, *N* (%)	13 (2.6)
Schizoaffective disorder, *N* (%)	81 (15.8)
Psychosis NOS, *N* (%)	31 (6.1)
Social contacts within previous week, median, IQR	2 (1–4)
Satisfaction with friendships (score 1–7), median, IQR	5 (4–6)

*N* = 511. Data is provided on complete cases

*Original, non *Z*-standardised values

In univariable negative binomial regression models of social contacts, people with psychosis living in areas with higher population density had fewer social contacts (relative risk [RR] = 0.88, 95% CI = 0.79–0.98, *p* = 0.02). No differences were found with regard to deprivation (Z-standardised RR = 0.98; 95% CI = 0.89–1.08, *p* = 0.73) or social fragmentation (Z-standardised RR = 0.98; 95% CI = 0.88–1.08, *p* = 0.69). Level of education, living alone, living in independent accommodation, being employed, and being white British were also associated (*p* < 0.01) with social contacts and hence were included in the multivariable modelling, along with population density. The length of illness did not show any association with the number of social contacts.

In multivariable models, the association between higher population density and lower number of social contacts remained after adjustment for participant-level characteristics (Z-standardised RR = 0.88; 95% CI = 0.79–0.99, *p* = 0.03; Table 2).

**Table 2:** Univariable and multivariable negative binomial regression models testing cross-sectional associations of number of social contacts in the previous week for each participant (outcome variable) with neighbourhood-level characteristics and participant-level characteristics

Independent variables	Univariable models			Multivariable models		
	RR*	CI** (95%)	p***	RR*	CI** (95%)	p***
Neighbourhood-level variables****						
Population density	0.88	0.79–0.98	0.02	0.88	0.79–0.99	0.03
Index of Multiple Deprivation	0.98	0.89–1.08	0.727			
Social Fragmentation Index	0.98	0.89–1.08	0. 69			
Participant-level characteristics						
Age (years)	1.00	0.99–1.01	0.39			
Gender (female versus male)	0.85	0.69–1.05	0.12			
Marital status (single vs not single)	1.05	0.83–1.32	0.69			
Tertiary or higher education (vs lower level of education)	0.82	0.67–1.00	0.05	0.87	0.71–1.06	0.17
Living alone (vs. living with others)	0.75	0.62–0.92	0.01	0.76	0.61–0.93	0.01
Living independently (vs. living in supervised settings)	0.80	0.63–1.01	0.06	0.90	0.71–1.15	0.42
Any employment (vs. not employed)	0.73	0.57–0.93	0.01	0.76	0.59–0.97	0.03
Receiving welfare benefits (vs. not receiving benefits)	1.06	0.59–1.89	0.85			
Years since first contact with services	1.000	0.99–1.01	0.93			
Born in the United Kingdom (vs. born abroad)	0.91	0.71–1.16	0.43			

*Relative risk

**Confidence interval

***Significance level set at *p* < 0.05

****Z-standardised

In univariable ordinal regression models of satisfaction with friendships, population density did not show a significant association with satisfaction with friendships (RR = 1.08; 95% CI = 0.93–1.26, *p* = 0.31). Social deprivation (Z-standardised RR = 1.13; 95% CI = 0 0.97–1.32, *p* = 0.13) and social fragmentation (Z-standardised RR = 1.15; 95% CI = 0.98–1.34, *p* = 0.08) also did not have significant associations with satisfaction with friendships. Hence, a multivariable model was not developed.

## Discussion

We found that people with psychosis living in more densely populated areas reported fewer social contacts, in contrast with results from similar studies in general populations [18–21]. Social deprivation and social fragmentation scores were not associated with the number of social contacts. Subjective satisfaction with friendships was not associated with any of the considered neighbourhood-level characteristics.

This study is the first to address the question of how neighbourhood-level variables were associated with social contacts in people with psychosis. We recruited a large sample across several mental health providers covering a variety of urban and rural areas in England. We considered several potential covariates, including the length of illness, which was not associated with either number of social contacts or satisfaction with friendships, and did not confound our results. The wide spread of 511 participants across 390 areas provided a high variance in neighbourhood characteristics with no clustering effect.

Our study has some limitations. First, selection bias might have influenced our results. It is possible that people who agreed to participate had different characteristics (i.e., they had more social contacts or were more satisfied with friendships) from those who declined to participate. Their relationships with clinicians who first approached them for participation might also might have made a difference as to whether they would accept or not. Moreover, whilst we have made efforts to recruit from both secondary and primary care services, we might not have reached people with psychotic disorders who are not engaging with either of these services or have not reached the threshold for their interventions. Associations between variables tend to be more robust towards selection bias than prevalence estimates [31], but we cannot exclude that a selection bias might have also affected associations (e.g., emphasising floor or ceiling effects of the variables). Second, the number of social contacts was self-reported and could have been affected by recall or desirability bias. Third, we excluded people who were unable to communicate in English due to inability to access specific interpretation services for the study or validated versions of the measures in all the different languages which would have been required. Fourth, the cross-sectional design of our study and the inclusion of participants with prevalent diagnoses meant we were unable to determine whether the observed association between higher population density and fewer social contacts was causal. Fifth, we did not measure the number of contacts occurring within mental health services. There could have been differences in service provision (e.g. presence or absence of day care initiatives) across the different sites involved which may have influenced the amount of social support that participants will have experienced. However, we felt that if we included social contacts as part of service activities our results would have been confounded by differences in service provision and we would not be able to accurately estimate the impact of neighbourhood variables on social contacts of participants. Finally, we did not have data on how long participants had lived at their current address. Future large, longitudinal studies are required to overcome these limitations.

As described above, our findings in a sample of people with psychotic disorders are in contrast with previous research in general populations [18–22]. Longitudinal designs are required to confirm our findings and test hypotheses as to how more densely populated environments might affect the social connections of people with psychosis. We could posit two hypotheses, which are linked to the concepts of “physical proximity” (access and opportunities for random interactions with social partners due to densely populated environments) and “psychological proximity” (sharing common interests from the outset or develops familiarity with) which were found to regulate social interactions in general populations [18, 20].

First, it is possible that people with psychosis are more likely to actively withdraw from social contacts in densely populated areas. Having a greater number of random social interactions may act as a stressor and exacerbate symptoms such as persecutory ideation or perceptual disturbances [32, 33]. Second, the causes for social isolation may relate to behaviours of other people towards those with psychotic disorders. Because opportunities for social interactions increase in more densely populated areas, people may become more socially selective (as they have greater choice) with whom they establish “psychological proximity” [34]. People with psychotic disorders may be viewed as less attractive social partners, especially if they have difficulty making conversation or with developing familiar relationships.

These hypotheses are not mutually exclusive. However, the lack of an association between population density in the area of residence and satisfaction with friendships might suggest a limited motivation of participants living in more densely populated areas to increase their social contacts. Moreover, our study did not identify evidence of an association between satisfaction with friendships and any of our three neighbourhood-level characteristics (population density, index of multiple deprivation, and social fragmentation index). It may be that neighbourhood-level characteristics are not as important as participant-level variables—for example, the severity of symptoms [35, 36]—in determining subjective feelings of dissatisfaction with one’s own social life.

Whatever the underlying reason, reduced social contacts and small social network size are linked to early mortality and morbidity in general populations [1, 2] and to negative social outcomes in psychosis [4]. Therefore, the association of higher population density and fewer social contacts in people with psychosis may be of high prognostic significance for this population, even in the absence of an effect on subjective feelings of satisfaction with friendships.

Clinicians in urban areas should be aware that their patients with psychosis are even more socially isolated than those who live in less densely populated areas, despite the arguably higher number of opportunities for socialisation.

Longitudinal studies over long period of time might help to confirm these findings and identify as to whether a change of residence (e.g., from an urban to a rural area) will be followed by a change in the number of social contacts.

These studies might inform interventions to reduce social isolation of people with psychosis which are currently being developed and tested [8, 37, 38]. They could also support policy decisions on housing programmes for people with psychosis who are socially isolated and have scarce family or other social support in the area in which they usually live.

Future studies should clarify why people with psychosis have fewer social contacts in areas with higher population density. This question could be addressed in the first instance by qualitative studies and requires replication and further exploration in larger longitudinal studies. Studies should explore the attitudes and behaviour of participants and of other people living in the same neighbourhoods, and evaluate changes over time to understand how social isolation develops and/or is maintained. These studies will be an important step towards the adaptation of social interventions and rehabilitation practices to the areas in which they are delivered.

It is hoped that these studies might also help us to understand better the complex pathways and factors that lead many patients with psychotic disorders to develop and experience social isolation.

## Supplementary Information

Below is the link to the electronic supplementary material.

Supplementary file1 (DOCX 22 KB)

## Data Availability

The data that support the findings of this study are available on request from the corresponding author, DG. The data are not publicly available due to the research governance requirements of protecting potentially identifiable data of participants from public access. Access is possible for research purposes, provided that a formal data sharing agreement is completed.
