# Supplementary material for: Neighbourhood characteristics and social isolation of people with psychosis: a multi-site cross-sectional study
Source: Soc Psychiatry Psychiatr Epidemiol. 2021 Nov 17;57(9):1907–15. doi: 10.1007/s00127-021-02190-x (PMC9375739; doi:10.1007/s00127-021-02190-x)
Supplement: Supplementary file 1 — Supplementary file1 (DOCX 22 KB) [file 127_2021_2190_MOESM1_ESM.docx]

**SOCIAL CONTACTS’ ASSESSMENT (SCA) – List of contacts (to be completed during the interview)**

*Please list all the people you have been in contact with in the last seven days and answer the following questions for each of them. For “being in contact” we mean that you can name them and have had a chat (more than just greeting) in the last week. Please do not include first degree relatives (parents, siblings, children), people you are living with, mental health professionals or people you work with (unless the contact took place outside of work).*

| **List of contacts**  **(initials)** | **Type of relationship**  **(1=friend, 2=partner, 3=acquaintance,**  **4=other, spec.)** | **On how many days, in the last week, have you been in face to face contact with him/her?** | **Was the meeting one to one or in a group?**   1. one to one   (b) group  (c) both | **If both, on how many days did you have one to one meeting(s)?** | **On how many days, in the last week, have you been in contact by voice or video call (using phone, skype or facetime, etc.)?** | **On how many days, in the last week, have you been in contact by social networking, e-mail or text message?** | **Can you talk to him/her about your personal feelings and worries?**   1. Yes 2. No | **Did you do something for him/her in the last week?**   1. Yes 2. No   **If yes, what?** | **Did he/she do something for you in the last week?**   1. Yes 2. No   **If yes, what?** |
| --- | --- | --- | --- | --- | --- | --- | --- | --- | --- |
|  |  |  |  |  |  |  |  |  |  |
|  |  |  |  |  |  |  |  |  |  |
|  |  |  |  |  |  |  |  |  |  |
|  |  |  |  |  |  |  |  |  |  |
|  |  |  |  |  |  |  |  |  |  |
|  |  |  |  |  |  |  |  |  |  |
|  |  |  |  |  |  |  |  |  |  |
|  |  |  |  |  |  |  |  |  |  |

**How many people have you had face to face contact with in the past week?**

**On how many days in the previous week have you had a face to face contact?**
